# Supplementary material for: HiCAT: a tool for automatic annotation of centromere structure
Source: Genome Biol. 2023 Mar 28;24:58. doi: 10.1186/s13059-023-02900-5 (PMC10053651; doi:10.1186/s13059-023-02900-5)
Supplement: Supplementary file 10 — Additional file 10. Review history. [file 13059_2023_2900_MOESM10_ESM.docx]

**1^st^ round**

**Reviewer 1**

Gao & Yang et al. describe HiCAT, a new algorithm and software implementation enabling automated annotation of nested tandem repeats, such as the alpha satellite Higher Order Repeats (HORs) found at all human centromeres and pericentromeres. Their method’s purpose is similar to the HORmon tool from Kunyavskaya et al., with several differences in approach and outcomes. Most importantly, HiCAT can detect Local Nested HORs (LN-HORs), which HORmon usually instead annotates as structural variants. HiCAT represents an important contribution to the growing but limited toolkit of methods needed to annotate newly assembled satellite DNA regions in both human and non-human genomes. The union of HORmon and HiCAT results can illuminate the organization and evolution of centromere-associated satellite DNA in ways that semi-manual annotation might miss, while enabling the study of these regions in an automated fashion in many genomes. I agree with the authors that the improvements and extensions of HiCAT that they proposed in the Discussion section would make the method even more useful, and I hope they will work to implement the detection of hybrid monomers and the use of raw reads as input. This is a useful and principled method, and I enthusiastically recommend this work for publication. The following suggested changes could be completed in the short term to make the work more impactful and understandable.

1) Ease of installation and use:

I was able to install the software from the HiCAT github page and apply it to the provided test data, but only after struggling to establish a python environment with the appropriate dependencies to support its proper execution. In the dependencies section of the github page, numpy is listed twice (and version 2.7.1 does not exist). I was not able to locate version 3.0.3 of networkx, which is listed as unreleased on their website. I would suggest that the authors simplify the installation process by, for example, providing a .yml file for an appropriate anaconda environment or creating a bioconda package, as done for Stringdecomposer and HORmon. This will make it practical for more users to install and run HiCAT, with less continued support required from the authors.

It would be helpful if command line arguments could be provided with flags (e.g. -o <output directory>), and if you had default settings for output directory (e.g. create a directory called “hicat_out” by default), python path (root by default), stringdecomposer path (root by default), and threads (all available CPUs by default). It would be helpful in the github documentation if you could provide an example command to run the software on the test data.

2) Validation on simulated edge cases:

Though not fully necessary, it would help to see HiCAT’s performance validated on simulated HOR sequences for which the ground truth is known. This would especially help in validating the method on edge cases at extremes not present in the human or Arabidopsis genomes but potentially present in other genomes, for example when the monomer size is much larger or smaller than 171 bp. This would also allow the authors to evaluate model performance while varying the divergence levels of monomers and HORs, as well as the length and abundance of LN-HORs.

3) Minor points:

**Figure 1b**) As illustrated, it appears that the region labeled as “Local Nested” could be explained by a single deletion event. This raises a larger question that could use some clarification in the text. If a HOR exists with the sequence ABCDEF and you observe the sequence ABABCDEF, would the HOR be annotated as AB~~CDEF-~~ABCDEF (i.e. two HORs, one with a structural variant due to a single deletion of BCDEF), or would it be annotated as (AB)*xn*CDEF? That is, what distinguishes a structural variant HOR from a LN-HOR? If the sequence were ABABABCDEF, then the tandem duplication of AB would seem more likely to be due to nested tandem repeat amplification rather than by a double deletion process (acknowledging that unequal exchange can cause apparent deletions and insertions in satellite DNA). Thus, there is an opportunity for the authors to clarify how HiCAT would classify a more diverse set of example evolutionary scenarios, and how this might differ from HORmon. A supplementary figure, with a schematic showing different hypothetical monomer arrangements and how HiCAT would classify them, would help an end user to interpret the results appropriately.

**Line 150**) It’s not immediately clear why the CEN15 R1L11 monomer indices go up to 14 (but 5, 12, 13 are missing). Adding a clause here to clarify this would help. Is the order of the unique monomer indices meaningful, or are the identifiers completely arbitrary? Would it be possible to re-order the monomer identifiers after inference of the most frequent HOR so that they appear in numerical order? This would help with readability and interpretation, making it easier to compare HORs and spot variants from the canonical monomer sequence. For example, CEN15 R1L11 would become (1-2-3-4)xn-5-6-7-8-9-10-11, with any remaining monomer classes taking on larger numbers. As another example, on line 162, renaming the identifiers 3-4-7-5-1-2 to 1-2-3-4-5-6 would make it easier to see that they are reversed in the inverted region of D1Z7. Although the phase of this ordering might still need to be arbitrary, having them be in the same order as found in the canonical HOR would improve readability.

**Line 164**) How does the reported D1Z7 6-mer HOR compare to the semi-manual annotation in the supplement of Altemose et al. 2022?

**Line 165**) A comparison and citation to Logsdon et al. 2021 should be added here (<https://doi.org/10.1038/s41586-021-03420-7>). They describe HORs in cen8 with differing numbers of monomers (4, 7, 8, 11) and location bias, as the authors describe here.

**Line 182**) The McNulty and Sullivan review does not report a 6-mer HOR in 1/5/19. Add citation to e.g. Uralsky et al. 2019 for this.

**Line 186**) How does the CEN5 R1L12 HOR compare to the semi-manual annotation in the supplement of Altemose et al. 2022?

**Figure 5a**) It is difficult to interpret this plot easily, as the desired comparison is between HiCAT and HORmon, not between Coverage and # Breakpoints. I would suggest breaking it up as done in Fig S5a-b (but overlaying all data points onto the boxplots). Alternatively, you could add a visual cue to the existing plot, such as a marker of “Ideal Annotation” in the upper left corner of the plot area, which would make it visually more obvious that HiCAT is outperforming HORmon by these metrics.

**Reviewer 2**

The basic unit of the human centromere, the alpha-satellite monomer, have many variants. A series of monomers are known to form higher order repeat structures (HORs), and are duplicated very frequently. While there are many possible ways to define HORs, no definitive method exists at this time, and this paper proposes such a method. Their efforts are to be appreciated; however, its algorithm is somewhat lousy, and results are described in a dry manner and lack biological findings.

I hope the following comments will help to improve the quality of the paper.

L109: "to optimize monomer inference 109 and HOR detection at the same time"

Mathematically describe the definition of the objective function of this optimization problem.

L110: "we applied a TR coverage maximization strategy to guide parameter selection"

No precise definition of this "maximization" strategy is found even in the method section on "mining HORs" from L340 to L387. It is unclear what the algorithm (in the method and supplementary document) really maximizes. The algorithm in "mining HORs" from L340 to L387 is ad hoc. No algorithmic analysis of the guarantees of the proposed algorithm has been performed.

L133: It is not presented precisely how to modify the similarity threshold t_d to obtain the next threshold t_{d+1}. This step is an important one that affects the output, but no serious description is found in the additional files.

L153: "most of them expanded twice"

L164: "most of them expanded four times (Fig. 3g)."

Can you verify that this expansion really happened evolutionarily? You can state that most of them have two copies.

L137-L169: These two paragraphs are descriptive; listing HOR patterns is somewhat tedious. Can you conclude any biological findings from these HOR patterns?

L179-L200: It is interesting to identify HORs with nested units such as R1L12, R2L14, and R1L14, but its fundamental units are well understood. It would be meaningful if you could have analyzed the functional consequences of these nested HORs. In its current form, no serious biological findings are given.

L215: It might be meaningful to reduce the number of annotation breakpoints and increase continuity. However, does it suggest any new model of centromere evolution? For examples in Figure 5c-e, the upper triangles showing similarities between HOR instances will be of interest to most researchers because it is very informative in understanding centromere evolution. To this end, HORmon HORs seem to be sufficient. It is unclear if there is any added value in your longer, nested HORs.

L286: The efficiency of a program is required, but the accuracy is more crucial. Since the definitions of optimization and maximization are not detailed, it is very difficult to evaluate the merits of the proposed algorithm.

**Authors’ response**

Reviewer #1

Gao & Yang et al. describe HiCAT, a new algorithm and software implementation enabling automated annotation of nested tandem repeats, such as the alpha satellite Higher Order Repeats (HORs) found at all human centromeres and pericentromeres. Their method’s purpose is similar to the HORmon tool from Kunyavskaya et al., with several differences in approach and outcomes. Most importantly, HiCAT can detect Local Nested HORs (LN-HORs), which HORmon usually instead annotates as structural variants. HiCAT represents an important contribution to the growing but limited toolkit of methods needed to annotate newly assembled satellite DNA regions in both human and non-human genomes. The union of HORmon and HiCAT results can illuminate the organization and evolution of centromere-associated satellite DNA in ways that semi-manual annotation might miss, while enabling the study of these regions in an automated fashion in many genomes. I agree with the authors that the improvements and extensions of HiCAT that they proposed in the Discussion section would make the method even more useful, and I hope they will work to implement the detection of hybrid monomers and the use of raw reads as input. This is a useful and principled method, and I enthusiastically recommend this work for publication. The following suggested changes could be completed in the short term to make the work more impactful and understandable.

RESPONSE: Thank you so much for your enthusiastically comments on our method. According to your valuable comments, we have simplified the HiCAT installation process and added simulation tests as well as revised our manuscript. A point-to-point response to each comment is provided as following.

1) Ease of installation and use:

I was able to install the software from the HiCAT github page and apply it to the provided test data, but only after struggling to establish a python environment with the appropriate dependencies to support its proper execution. In the dependencies section of the github page, numpy is listed twice (and version 2.7.1 does not exist). I was not able to locate version 3.0.3 of networkx, which is listed as unreleased on their website.

I would suggest that the authors simplify the installation process by, for example, providing a .yml file for an appropriate anaconda environment or creating a bioconda package, as done for Stringdecomposer and HORmon. This will make it practical for more users to install and run HiCAT, with less continued support required from the authors.

RESPONSE: Thank you for pointing out this issue. We apologize for unclear version of HiCAT dependencies. The version of numpy is 1.22.3 and networkx is 2.7.1. We also clarified the version of other dependent package in the README

(https://github.com/xjtu-omics/HiCAT). To simplify the installation process, we integrated StringDecomposer into HiCAT by copying the source code to HiCAT (./stringdecomposer) and provided two installation options:

 Install from source code and running

Step 1: conda install -y --file requirements.txt

Step 2: cd ./stringdecomposer && make

python HiCAT.py -i ./testdata/cen21.fa -t ./testdata/AlphaSat.fa

 Install from conda environment and running

conda install -c xjtuomics hicat

hicat -i ./testdata/cen21.fa -t ./testdata/AlphaSat.fa

We have updated our Github (https://github.com/xjtu-omics/HiCAT).

It would be helpful if command line arguments could be provided with flags (e.g. -o and if you had default settings for output directory (e.g. create a directory called “hicat_out” by default), python path (root by default), stringdecomposer path (root by default), and threads (all available CPUs by default). It would be helpful in the github documentation if you could provide an example command to run the software on the test data.

RESPONSE: Thank you for your kind suggestion. We have revised the code of HiCAT and set the flags for each argument. We updated the HiCAT.py file. The revised help page of HiCAT likes Fig. R1.

The revised version of HiCAT do not need user input python path and stringdecomposer path. User only need to provide DNA sequence (-i) and monomer template sequence (-t) with default parameters. Following your suggestion, we revised HiCAT to creates a directory called “HiCAT_out” by default and provide -o parameter for user to create their own output directory. We provided an example command to run the software on the test data which is

python HiCAT.py -i ./testdata/cen21.fa -t ./testdata/AlphaSat.fa

2) Validation on simulated edge cases:

Though not fully necessary, it would help to see HiCAT’s performance validated on simulated HOR sequences for which the ground truth is known. This would especially help in validating the method on edge cases at extremes not present in the human or Arabidopsis genomes but potentially present in other genomes, for example when the monomer size is much larger or smaller than 171 bp. This would also allow the authors to evaluate model performance while varying the divergence levels of monomers and HORs, as well as the length and abundance of LN-HORs.

RESPONSE: Thanks for your valuable suggestions. To comprehensively evaluate HiCAT performance, we simulated two datasets including canonical HORs and LN- HORs. The simulation process contains two steps: simulating monomer sequences and simulating DNA sequences (Fig. R2a). In the first step, we set HOR unit with five monomers and simulated 40 HOR units as canonical HOR monomer sequences. Next, we randomly selected 20 out of 40 canonical HOR units to generate LN-HORs. For each chosen unit, we randomly selected one to four consecutive monomers of the unit and tandemly amplified the chosen monomer(s) for a random number of times within the range from two to five. In the second step, we simulated raw monomer template DNA sequence with 100, 200, 300 and 400bp since satellite monomer size are often observed in 100-400bp (Talbert et al. 2020, Exp Cell Res, PMID: 32035948), respectively. Then, we generated five diverged monomer templates by randomly mutating 10%, 20% or 30% bases for each raw monomer template. Finally, based on the monomer sequences acquired in first step, we generated final DNA sequences by randomly mutating 0.5%, 1.5% or 2.5% bases for each diverged monomer template (Fig. R2b, c). In each case, we can obtain 36 (4×3×3) sets of simulated data and we repeated 10 times. Finally, we obtained 360 sets of canonical HOR data and 360 of LN- HOR. The code for generating simulation data is in https://github.com/xjtu- omics/HiCAT.

We first evaluated and compared HiCAT with HORmon (using default parameter) based on simulated canonical HORs. We found that the number of HORs annotated by HiCAT is closer to the ground truth of 40 HOR units than that of HORmon regardless of monomer size, monomer divergence, and HOR divergence (Fig. 3 a, b, c and Supplementary table S1). Specifically, HiCAT correctly annotated 40 HOR units in 96.11% (346/360) simulated canonical HORs while 61.67% (222/360) for HORmon (Supplementary table S2). We investigated the special cases that HORmon failed, and found that HORmon misannotated majority of the events (89 out of 90 as 33 HOR units) while HiCAT correctly annotated most of the events (82 out of 90 as 40 HOR units) when monomer size was 400bp, regardless of monomer divergence and HOR divergence (Fig. 3a and Supplementary table S2). Next, we evaluated HiCAT performance on simulated LN-HORs. On average, HiCAT annotated 19.7 local-nested units and 19.9 canonical units (Fig. 3d). Specifically, 90.56% (326/360) LN-HORs from HiCAT annotation were identical to the ground truth of 20 canonical HOR units and 20 LN-HOR units (Supplementary table S2). We further investigated the annotation results of HORmon on the simulated LN-HORs and found that HORmon annotated 11.1 single monomers, 38.5 partial HOR units and 39.3 canonical HOR units for each simulated LN-HOR dataset on average (Fig. 3d).

We have revised our manuscript by adding new paragraphs in Results (Section of “Performance evaluation on simulated datasets”) and Methods (Section of “Simulated tests”). We also provided new Fig. 3, Supplementary table S1 and S2 and adding Fig. R2 as Supplementary figure S3.

Figure R2| Building simulated datasets. a. The process for simulated datasets. The grey rectangle represents raw monomer template. We simulated different templates with 100, 200, 300 and 400bp. Different colored rectangles represent different monomers in HORs. The monomer divergence between diverged monomer templates are 10%, 20% and 30%. The divergence between final monomers in monomer sequence and diverged monomer templates are 2.5%, 1.5%. 0.5%. b. An example dotplot of simulated canonical HORs with monomer size 200bp, monomer divergence 10% and HOR divergence 0.5%. c. An example dotplot of simulated local nested HORs with monomer size 200bp, monomer divergence 10% and HOR divergence 0.5%.

3) Minor points:

- Figure 1b) As illustrated, it appears that the region labeled as “Local Nested” could be explained by a single deletion event.

This raises a larger question that could use some clarification in the text. If a HOR exists with the sequence ABCDEF and you observe the sequence ABABCDEF, would the HOR be annotated as ABCDEF-ABCDEF (i.e. two HORs, one with a structural variant due to a single deletion of BCDEF), or would it be annotated as (AB)xnCDEF? That is, what distinguishes a structural variant HOR from a LN-HOR? If the sequence were ABABABCDEF, then the tandem duplication of AB would seem more likely to be due to nested tandem repeat amplification rather than by a double deletion process (acknowledging that unequal exchange can cause apparent deletions and insertions in satellite DNA).

Thus, there is an opportunity for the authors to clarify how HiCAT would classify a more diverse set of example evolutionary scenarios, and how this might differ from HORmon. A supplementary figure, with a schematic showing different hypothetical monomer arrangements and how HiCAT would classify them, would help an end user to interpret the results appropriately.

RESPONSE: Thanks for your comment. Currently, HiCAT annotates both canonical and local-nested HORs (LN-HORs) while the latter was defined as tandem amplification of a subunit (Fig. R3), such as from ABCDEF (canonical) to (AB)×nCDEF (local-nested). For the special case of AB-ABCDEF, HiCAT annotates it as (AB)x2CDEF while a single deletion of CDEF indeed results in the same outcome as you suggested (Fig. R3b, c). We admit that in this case, both interpretations are equivalent. However, we observed abundant cases with n > 2, like ABABABCDEF.

For example, in CEN1, two monomers 1-2 in HOR pattern 1-2-5-6-4-3 is tandem amplification and most of them have four copies (Fig. 4g). We also detected other patterns of 1-2-5-6-4-3 with different copy numbers of 1-2 even more than ten (Fig. 4g). As you proposed, these cases would seem more likely to be due to nested tandem repeat amplification rather than by a multiple deletion process (Fig. R3d, e). Having observed above instances, we believe that a unified framework of tandem repeat amplification to annotate both n = 2 and n > 2 would be preferred, but we do not rule out alternative explanations, like deletions. We updated Figure 1b to illustrate a typical local nested event with n = 4 in CEN9. And we also revised our manuscript by adding these clarifications in Discussion section (line 356-370) and adding Fig. R3 as Supplementary figure S10 based on your examples.

Fig. R3. The different hypothetical monomer arrangements in HiCAT annotation. a. All HOR units are canonical. b. A part of HOR unit AB duplicated. c. Hypothetical monomer arrangements with tandem duplication and deletion for duplication. d. A part of HOR unit AB triplicated. Differently colored rectangles represent different monomer annotation. e. Hypothetical monomer arrangements with tandem duplication and deletion for triplication.

As for the difference between HiCAT and HORmon to annotate diverse loci, HORmon detects a single cycle in a de Bruijn graph constructed based on monomer sequences as HOR and breaks the monomer connections absent from the cycle to classify canonical and partial HORs. HORmon's strategy is efficient but do introduce the breakage of HOR cycle wherever local-nested events appear. HiCAT is designed based on hierarchical compressed tandem repeats strategy, which is well equipped to model the evolution of centromere due to the following two reasons. First, tandem amplification is the typical characteristics of centromere DNA sequence. Second, it is generally accepted that each human centromere evolves from a single ancestral HOR (Kunyavskaya et al. 2022, Genome Research, PMID: 35545449). We show an example of HORmon and HiCAT annotation in CEN1 with monomer sequence 1-2-1-2-5-6-4- 3-1-2-1-2-1-2-1-2-5-6-4-3 (Fig. R4). HiCAT annotated this monomer sequence as two LH-HOR units. However, HORmon reported two canonical HORs, two partial HORs, two single monomers (not belonging to any HORs) and even two regions without annotation (Fig. R4).

Fig. R4. Comparison between HiCAT and HORmon annotation in an example locus of human CHM13 CEN1 (chr1:121,948215-121,951,615).

- Line 150) It’s not immediately clear why the CEN15 R1L11 monomer indices go up to 14 (but 5, 12, 13 are missing). Adding a clause here to clarify this would help. Is the order of the unique monomer indices meaningful, or are the identifiers completely arbitrary? Would it be possible to re-order the monomer identifiers after inference of the most frequent HOR so that they appear in numerical order? This would help with readability and interpretation, making it easier to compare HORs and spot variants from the canonical monomer sequence. For example, CEN15 R1L11 would become (1- 2-3-4)xn-5-6-7-8-9-10-11, with any remaining monomer classes taking on larger numbers. As another example, on line 162, renaming the identifiers 3-4-7-5-1-2 to 1-2- 3-4-5-6 would make it easier to see that they are reversed in the inverted region of D1Z7. Although the phase of this ordering might still need to be arbitrary, having them be in the same order as found in the canonical HOR would improve readability.

RESPONSE: Thanks for pointing out this. In previous version of HiCAT, the monomer IDs are completely arbitrary. Now, we have updated the source code of HiCAT to rename the monomers. In the updated version of HiCAT, we re-order the monomer ID based on R1 HOR (the first one) and the monomers not in R1 HOR will be re-named based on the frequency of occurrence. For example, in CEN15, the new ID is 1-2-3-4-5-(6-7-8-9)×n-10-11 We revised all figures, tables and manuscript.

- Line 164) How does the reported D1Z7 6-mer HOR compare to the semi-manual annotation in the supplement of Altemose et al. 2022?

RESPONSE: The reported D1Z7 6-mer HOR is the same as Altemose et al. 2022 annotation. And in the study of Altemose et al. 2022, they reported a 1.7Mb inversion in D1Z7, which is also detected by HiCAT (Fig. 4f).

- Line 165) A comparison and citation to Logsdon et al. 2021 should be added here (https://doi.org/10.1038/s41586-021-03420-7). They describe HORs in cen8 with differing numbers of monomers (4, 7, 8, 11) and location bias, as the authors describe here.

RESPONSE: Thanks for your kind suggestion. We cannot find the detailed HOR annotation in the supplementary materials of Logsdon et al. 2021. So, we compared monomer and HOR annotation with the results from Altemose et al. 2022 which is also based on CHM13 genome. We revised our manuscript in Results (Section of “Overall performance for human CHM13 centromeres”) and added the citation of Logsdon et al. 2021 and the comparison results. We also added a Supplementary figure S5 to show the comparison in monomer and HOR annotation.

- Line 182) The McNulty and Sullivan review does not report a 6-mer HOR in 1/5/19. Add citation to e.g. Uralsky et al. 2019 for this.

RESPONSE: Thank you for reminding us. We have revised the citation.

- Line 186) How does the CEN5 R1L12 HOR compare to the semi-manual annotation in the supplement of Altemose et al. 2022?

RESPONSE: In HiCAT annotation, R1L12 contains 12 monomers. CEN5 contains hybrid monomers and D5Z2 is the result of dehybridization. We found that monomer annotation from HiCAT was consistent with that from Altemose et al. 2022 (Fig. R5). Three major hybrid monomers S1C1/5/19H1L.2/6, S1C1/5/19H1L.6/4 and S1C1/5/19H1L.2/4 were defined as monomer 2, 7 and 8 in HiCAT, respectively (Fig. R3).

We revised our manuscript in section of Substantial differences between HiCAT and semi-manual HOR annotations in CEN5 and CEN17 and adding the Fig. R5a and b as Supplementary figure S6b and c.

Figure R5. HiCAT annotation compared with Altemose et al. 2022. a. Monomer Sankey plot for CEN5 showing the high consistency between HiCAT and Altemose et al. 2022. To display the frequent monomers, we filtered the links with fewer than 10 matches. b. Compared HOR pattern in HiCAT and Altemose et al. 2022.

- Figure 5a) It is difficult to interpret this plot easily, as the desired comparison is between HiCAT and HORmon, not between Coverage and # Breakpoints. I would suggest breaking it up as done in Fig S5a-b (but overlaying all data points onto the boxplots). Alternatively, you could add a visual cue to the existing plot, such as a marker of “Ideal Annotation” in the upper left corner of the plot area, which would make it visually more obvious that HiCAT is outperforming HORmon by these metrics.

RESPONSE: Thank you for your kind advice. We revised the Fig. 6 (Fig. 5 in previous version) and Supplementary figure S7 (Supplementary figure S5 in previous version) followed your suggestions.

Reviewer #2 (Remarks to the Author):

The basic unit of the human centromere, the alpha-satellite monomer, have many variants. A series of monomers are known to form higher order repeat structures (HORs), and are duplicated very frequently. While there are many possible ways to define HORs, no definitive method exists at this time, and this paper proposes such a method. Their efforts are to be appreciated; however, its algorithm is somewhat lousy, and results are described in a dry manner and lack biological findings.

I hope the following comments will help to improve the quality of the paper.

RESPONSE: Thanks for your comments. A point-to-point response is provided as following.

Comment 1. L109: "to optimize monomer inference 109 and HOR detection at the same time"

Mathematically describe the definition of the objective function of this optimization problem.

RESPONSE: We apologize for the unclear description. We apply brute force enumeration rather than optimization procedure in HiCAT. Here, we built a series of block graphs based on the similarity threshold iteratively increasing from the minimum value (by default 94%) to nearly 100% with a specific step (by default 0.5%) and obtained all monomer inference and HOR annotation results. Finally, to build interconnections between monomer inference and HOR detection, we calculated the associated HOR coverage for each similarity threshold and chose the threshold with the largest coverage to obtain HiCAT HOR annotation.

We have revised the description in our manuscript by removing the confusing terms like, “maximization” and “optimize” and revised Figure 2 by updating “Select the maximize tandem repeat coverage result” to “Select the similarity threshold leading to the largest tandem repeat coverage”.

Comment 2. L110: "we applied a TR coverage maximization strategy to guide parameter selection"

No precise definition of this "maximization" strategy is found even in the method section on "mining HORs" from L340 to L387. It is unclear what the algorithm (in the method and supplementary document) really maximizes. The algorithm in "mining HORs" from L340 to L387 is ad hoc. No algorithmic analysis of the guarantees of the proposed algorithm has been performed.

RESPONSE: Thanks for pointing out this. As we explained in your comment #1, the maximization strategy is defined as the selection of the similarity threshold with the largest HOR coverage after a series of block graph processing.

To avoid the confusing term “maximization”, we revised the section of "Overview of HiCAT" by adding the sentence “To build interconnections between monomer inference and HOR detection, we calculated the associated HOR coverage of each similarity threshold and chose the threshold with the largest coverage to obtain HiCAT HORs” in line 122-124.

Comment 3. L133: It is not presented precisely how to modify the similarity threshold t_d to obtain the next threshold t_{d+1}. This step is an important one that affects the output, but no serious description is found in the additional files.

RESPONSE: As we describe in our manuscript and your comment #1, the similarity threshold iteratively increasing from the minimum value to nearly 100% with a specific step (default 0.5%). In our research, based on a previous study in human centromeres (Bzikadze et al, 2020, Nature biotechnology, PMID: 32665660). we set the minimum similarity threshold as 94% since the similarity between HOR units was reported to be in the range of 95-100% in human.

For example, in Fig. 2,0 94%t = and1 0 0.5% 94.5%t t= + = . We revised the description in in Figure 2 legend by adding detail explanation of the modifying the similarity threshold.

Comment 4. L153: "most of them expanded twice"

L164: "most of them expanded four times (Fig. 3g)."

Can you verify that this expansion really happened evolutionarily? You can state that most of them have two copies.

RESPONSE: Thanks for your kind advice. We revised our manuscript by updating the state like "most of them expanded twice" to "most of them have two copies".

Comment 5. L137-L169: These two paragraphs are descriptive; listing HOR patterns is somewhat tedious. Can you conclude any biological findings from these HOR patterns?

RESPONSE: In these two paragraphs, we compared the HiCAT annotation with previous semi-manual inference in human centromeres to demonstrate our automated annotation was largely consistent with previous semi-manual studies. From these HOR patterns detected by HiCAT, we concluded that HOR patters in human centromeres are diverse and some of them show location specificity. Moreover, the results showed that large structure rearrangements exist in some centromeres.

The importance of HiCAT to the community is appreciated by Reviewer #1 “HiCAT represents an important contribution to the growing but limited toolkit of methods needed to annotate newly assembled satellite DNA regions in both human and non-human genomes. The union of HORmon and HiCAT results can illuminate the organization and evolution of centromere-associated satellite DNA in ways that semi- manual annotation might miss, while enabling the study of these regions in an automated fashion in many genomes”. Due to the lack of effective tools and high- quality centromere assemblies to decode the centromere patterns, the evolutionary mechanisms of centromere within and among species are largely unexplored. HiCAT provides automated and generalization frameworks to handle the emergence of large- scale high-quality genome assemblies, enabling the investigation of centromere evolution.

We prepared this manuscript as a “Method” article, which “need not necessarily provide novel biological insights” according to the submission guided of Genome Biology. Nevertheless, we tried our best to illustrate biological findings in our manuscript with available data.

Comment 6. L179-L200: It is interesting to identify HORs with nested units such as R1L12, R2L14, and R1L14, but its fundamental units are well understood. It would be meaningful if you could have analyzed the functional consequences of these nested HORs. In its current form, no serious biological findings are given.

RESPONSE: In these paragraphs, we investigated the fine structures and organization of HOR units with length variation. In CEN5, we found the location bias of R1L12 (1- 2-3-4-5-6-1-7-(1-2-3-8)×n) three patterns (n=1, 2, 3). The canonical HORs (n=1) in R1L12 were significant enriched (p < 0.05, z-test) in the marginal area and LN-HORs (n=2 and n=3) were significantly enriched (p < 0.05, z-test) in the centre (Fig. 5c). In CEN17, we found that previous reported HOR D17Z1 was a special case of HiCAT R1L14 (1-2-3-4-5-6-(7)×n-8-9-10-11-12-13-14) with n=3 (Fig. 5e). And we also detected monomer 7 with different number of copies up to five (Supplementary figure S6g).

As we explained in your comment #5, HiCAT is a tool to detect the various different HOR patterns and we have demonstrated its performance. For serious biological findings required to be studied by a large sample project and more data in different level. We think that users apply HiCAT to their own data will result in many serious biological findings.

Comment 7. L215: It might be meaningful to reduce the number of annotation breakpoints and increase continuity. However, does it suggest any new model of centromere evolution? For examples in Figure 5c-e, the upper triangles showing similarities between HOR instances will be of interest to most researchers because it is very informative in understanding centromere evolution. To this end, HORmon HORs seem to be sufficient. It is unclear if there is any added value in your longer, nested HORs.

RESPONSE: Thanks for your comment. In our manuscript, we proposed a HOR annotation method and tool HiCAT, and demonstrated its performance based on simulated data, human CHM13 complete genome, and Arabidopsis thaliana Col-CEN complete genome. Although we did not propose new model of centromere evolution which require addition species genomes with centromere sequences, we detected many fine HOR structures in centromere and reported the detailed information of local-nested in HORs.

We agree that the triangle heatmaps showing similarities between HOR instances will be of interest to most researchers and reveal the general characters at a larger scale. In addition, HOR analysis is important and it can discover fine and high resolution landscapes like the study of Altemose et al. (Altemose et al. 2022, Science, PMID: 35357911), in which they analyzed the HOR landscapes in CHM13 centromeres and uncovered a strong relationship between the position of the centromere and the evolution of the surrounding DNA through layered repeat expansions. All of these indicate that the value of tools like HiCAT to be the foundation of further analysis of HOR annotation results, exploring the centromere evolutionary models. HORmon is one of the pioneer tools to annotate centromere, but it fails when monomer size is large, e.g. 400bp, as we showed in simulation tests, which might limit its application (Fig. 3). Moreover, as we showed in Fig. R4, HORmon breaks the LN- HORs into fragmented partial HORs, hindering the detection of fine structures and organization of HOR units with length variation in centromere. For example, in human centromere, we found that nested tandem amplification of dimer is common in CEN1 at a peak of four copies (Fig. 4g). In CEN5, we found that LN-HORs were enriched in the centre and canonical HORs were significant enriched in the marginal area (Fig. 5c). In CEN17, HiCAT reported monomer 7 with different numbers of copies up to five (Supplementary figure S6g). These longer and continuous HOR patterns were absent in HORmon annotation.

We have revised our manuscript in Discussion section (line 337-345) and clearly described the added value in our longer LN-HOR annotation.

Comment 8. L286: The efficiency of a program is required, but the accuracy is more crucial. Since the definitions of optimization and maximization are not detailed, it is very difficult to evaluate the merits of the proposed algorithm.

RESPONSE: As we describe in your comment #1, we apologize for the unclear description. We apply brute force enumeration rather than optimization procedure in HiCAT. We have revised the description in our manuscript by removing the confusing terms like, “maximization” and “optimization”.

We provide time analysis of all scenarios in discussion section to show the efficiency of HiCAT which can work well in DNA sequence ranging from 331Kb to 4.8Mb. Moreover, we added the simulation tests (in Results, section of “Performance evaluation on simulated datasets”) to compare HiCAT and HORmon with default parameters. The results demonstrated that HiCAT outperformed HORmon in terms of monomer size, HOR divergence and monomer divergence (Fig. 2), indicating the accuracy of HiCAT on HOR annotation

**2^nd^ round**

**Reviewer 1**

The authors went above and beyond to address my comments. The revised manuscript is greatly improved and suitable for publication.
